# Angle Class II, subdivision, with agenesis of mandibular second molars and extrusion of maxillary second molars^*^

**DOI:** 10.1590/2176-9451.20.2.110-118.bbo

**Published:** 2015

**Authors:** Rubens Rodrigues Tavares

**Affiliations:** 1 Specialist in Orthodontics and Facial Orthopedics, Universidade Paulista (UNIP). Certified by the Brazilian Board of Orthodontics and Dentofacial Orthopedics (BBO)

**Keywords:** Angle Class II malocclusion, Partial anodontia, Corrective Orthodontics, Dental implant

## Abstract

This clinical case reports the treatment of an Angle Class II malocclusion in a young woman with a balanced face affected by agenesis of second and third mandibular molars and subsequent extrusion of second maxillary molars. The atypical and peculiar occlusal anomaly led to individualized treatment proposed in order to normalize dental malpositions, with subsequent rehabilitation of edentulous areas by means of a multidisciplinary approach. This case was presented to the Brazilian Board of Orthodontics and Dentofacial Orthopedics (BBO) in partial fulfillment of the requirements for obtaining the title of certified by the BBO.

## Abstract (pt)

O presente caso clínico relata o tratamento de uma má oclusão de Classe II de Angle, em uma jovem com face harmoniosa, porém agravada por agenesias de segundos e terceiros molares inferiores e consequente extrusão dos segundos molares superiores. A anomalia oclusal atípica e peculiar levou a uma proposta de tratamento individualizada, visando normalizar os maus posicionamentos dentários e uma posterior reabilitação das áreas edêntulas, por meio de uma abordagem multidisciplinar. O presente caso foi apresentado à Diretoria do Board Brasileiro de Ortodontia e Ortopedia Facial (BBO), como parte dos requisitos para a obtenção do título de Diplomado pelo BBO.

## INTRODUCTION

A female patient presented for initial examination at the age of 14 years and three months and was found to be in good general health. No significant information was found in her past medical and dental records. She did not have, nor did she report having, any deleterious oral habits. As chief complaint she reported that some mandibular teeth were missing, which resulted in the presence of spaces, rotations and difficulty chewing in the posterior region. She had little growth potential, as she reported that her menarche had occurred when she was about 12 years old. While in many subjects the hereditary component is involved in determining partial anodontia, this aspect was not investigated in this case.

## DIAGNOSIS

**Figure 1 -:**
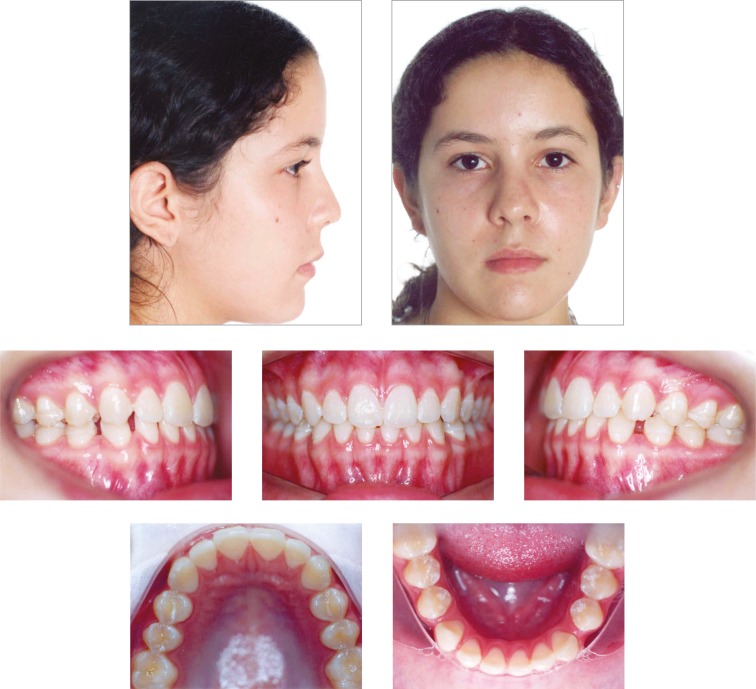
Initial facial and intraoral photographs.

She had a rather well-balanced mesofacial pattern without any serious neuromuscular functional changes, as well as a slightly convex profile and slightly protrusive maxillary and mandibular lips (UL-S line = 3mm LL-S line = 2 mm). This feature seemed fully compatible with the patient's age group (Fig 1 and Table 1).

**Table 1 -:** Initial (A) and final (B) cephalometric values.

	Measurements		Normal	A	B	Dif. A/B
Skeletal pattern	SNA	(Steiner)	82°	78°	78°	0
	SNB	(Steiner)	80°	76°	76.5°	0.5
	ANB	(Steiner)	2°	2°	1.5°	0.5
	Angle of convexity	(Downs)	0°	2°	1°	1
	Y axis	(Downs)	59°	61°	62°	1
	Facial angle	(Downs)	87°	87°	87°	0
	SN-GoGn	(Steiner)	32°	31°	30°	1
	FMA	(Tweed)	25°	26°	24°	2
Dental pattern	IMPA	(Tweed)	90°	85°	89°	4
	1.NA (degrees)	(Steiner)	22°	17°	18°	1
	1-NA (mm)	(Steiner)	4 mm	3.5 mm	2 mm	1.5
	1.NB (degrees)	(Steiner)	25°	20°	20°	0
	1-NB (mm)	(Steiner)	4 mm	3.5 mm	3 mm	0.5
	- Interincisal angle	(Downs)	130°	145°	147°	2
	1-APo	(Ricketts)	1 mm	0.5 mm	0 mm	0.5
Profile	Maxillary lip — S-line	(Steiner)	0 mm	3 mm	2.5 mm	0.5
	Mandibular lip — S-line	(Steiner)	0 mm	2 mm	1 mm	1

**Figure 2 -:**
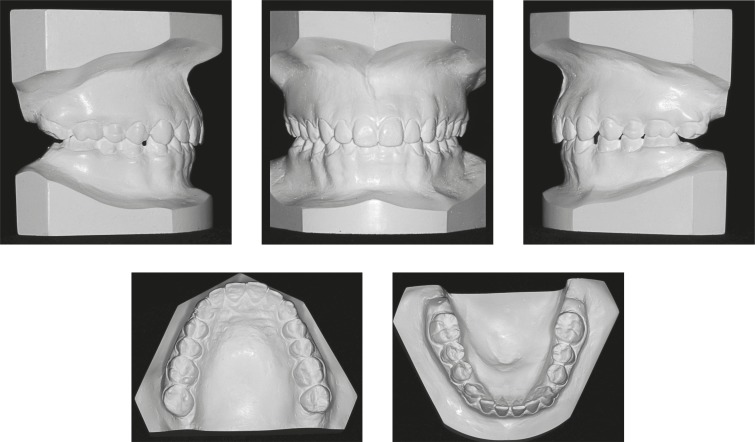
Initial casts.

Dental analysis (Figs 1 and 2) disclosed Angle Class II malocclusion, subdivision right, aggravated by the absence of second and third mandibular molars, distal migration of mandibular posterior teeth, and extrusion of second maxillary molars. In addition to the aforementioned teeth, tooth #18 was also missing. She presented asymmetry of maxillary canines in the anteroposterior direction and no coincidence between maxillary and mandibular midlines and the midsagittal plane. The maxillary midline was shifted to the left while the mandibular one was shifted to the right.^1^^,^ 2 She had an increased overbite with sharp incisal disocclusion and well-adjusted anterior centric stop. In the mandibular dental arch, there was generalized diastema, pronounced in the region between canines and first premolars.

**Figure 3 -:**
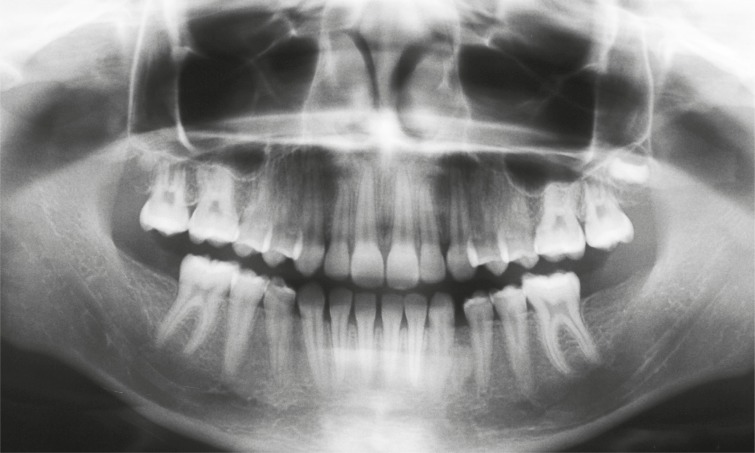
Initial panoramic radiograph.

Panoramic radiograph (Fig 3) revealed good root formation of all teeth, in addition to absence of teeth #37 and 47. As regards third molars, it was observed that only tooth #28 was going through early stages of formation, about Nolla stage 4, with all other teeth missing.

**Figure 4 -:**
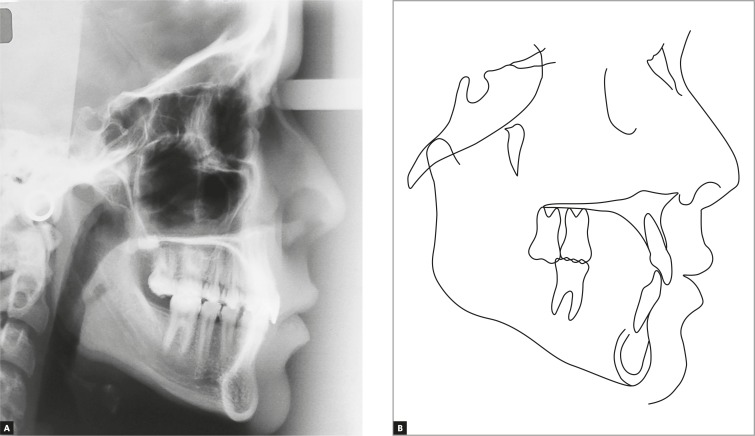
Initial profile cephalometric radiograph (A) and cephalometric tracing (B).

Profile cephalometric radiograph and cephalometric tracing (Fig 4) revealed good maxillomandibular relationship in the vertical (SN-GoGn = 31^o^; FMA = 26^o)^, and anteroposterior direction, with Class I skeletal pattern (SNA = 78^o^; SNB = 76^o^; ANB = 2^o)^. Maxillary and mandibular incisors were slightly upright (1.NA = 17^o^; 1.NB = 20^o)^, thereby increasing the interincisal angle (1/1 = 145^o)^. These and other cephalometric values ​​are shown in Table 1.

## TREATMENT PLAN

**Figure 5 -:**
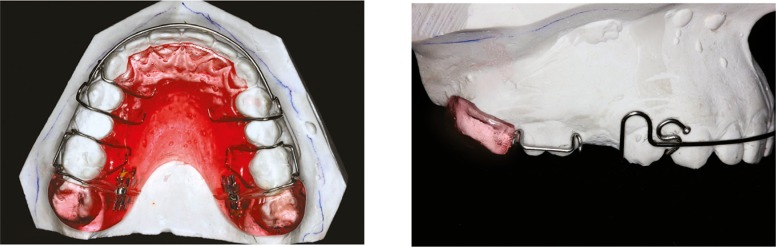
Occlusal and right lateral views of the removable orthodontic appliance.

Due to dental asymmetry, treatment planning aimed to produce a distal movement of maxillary molars on the right side and, at the same time, maintain vertical control of maxillary second molars already extruded due to absence of antagonists. This could allow retraction of the maxillary right quadrant so as to correct the anteroposterior asymmetry of canines and gain space to correct the deviation in the maxillary midline. As anchorage, one alternative would be to use mini-implants, which would allow a more effective control of distalization of maxillary teeth. However, the patient's legal guardians rejected this alternative, perhaps because it was not popular at that time. A removable appliance was therefore used encapsulating teeth #17 and 27 to prevent extrusion, along with an expansion screw for distalization (Fig 5). A hook was also placed on the right side to deploy Class II mechanics as soon as the mandibular arch had been leveled. Thereafter, a fixed orthodontic appliance would be placed with a stop on the already distalized posterior teeth, and mechanics applied to retract tooth #13, thereby achieving symmetry with its antagonist and space for midline correction.

In the mandibular arch, the fixed orthodontic appliance would allow not only the leveling of the occlusal plane, a necessary step to correct severe overbite, but also the mesialization of posterior teeth, especially on the right side, to correct the most distal position of the right canine relative to the left. As a result, diastemata would be eliminated, providing ideal canine and first molar occlusion and adjusting the spaces for rehabilitation with dental implants osseointegrated in the region of second molars, in addition to correcting the mandibular midline.

## TREATMENT PROGRESS

Treatment began eight months after completion of the initial examination, when the patient was almost 15 years old. This waiting time was meant to postpone, albeit slightly, the completion of treatment, bringing it a little closer to the end of patient's overall growth, when other rehabilitation resources, including dental implants, would be available.

For the maxillary arch, a removable orthodontic appliance was fabricated and installed.^3^ It consisted of a Hawley retainer in the anterior region, Adams clasps on the first molars and bilateral screws to distalize teeth #17 and 27 (Fig 5). These teeth were kept encapsulated in the acrylic to prevent further extrusion during distalization. The appliance also featured a hook on the right side for Class II elastics. The patient was instructed to wear the appliance full time, removing it only to eat, engage in extreme sports and learn foreign languages. The recommended activation was 1/4 of a turn, in each screw, every five days. To ensure better vertical control, the maxillary second molars were replenished with self-curing acrylic resin every six weeks.

Orthodontic bands were placed on the mandibular first molars, and Roth prescription brackets with 0.018 x 0.030-in slots were bonded to all other teeth. Alignment and leveling were then achieved using up to 0.016-in round stainless steel archwires. Class II elastics were thereafter introduced to be worn on the right side, anchored on the removable appliance.

After creating spaces between first and second molars, maxillary fixed orthodontic appliance (Roth prescription, 0.018 x 0.030-in slot) was bonded after alignment and leveling, using the same sequence of round stainless steel archwires. All teeth received a mesial stop after distalization to progressively move first molars, premolars and canines distally; more so on the right side, to ensure symmetry between homologous teeth. A 0.016 x 0.022-in TMA archwire with T loops was used to intrude and level the second molars.

**Figure 6 -:**
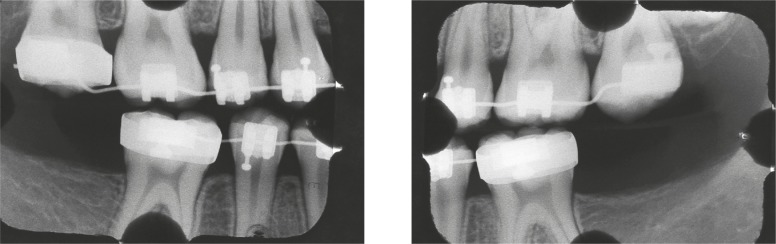
Bite-wing radiograph. Note intrusive step on the archwire in teeth #17 and 27.

Then, 0.016 x 0,016-in and 0.016 x 0,022-in Elgiloy archwires were used for both maxillary and mandibular arches, while intrusive steps^4^were incorporated to second molars (Fig 6). At this treatment stage, Class II elastics were used bilaterally to finish the relationship between molars and canines in an ideal occlusion. After obtaining the interocclusal space needed for rehabilitation with dental implants osseointegrated in the region of mandibular second molars, the appliance was kept passive. It is noteworthy that implant surgery was delayed by about six months in order to make it coincide, as much as possible, with the end of patient's growth. After the osseointegration period, the prosthetic phase was performed concurrently with the removal of the fixed orthodontic appliance.

A removable plate with a Hawley retainer was used for retention in the maxillary arch in the anterior region, and a fixed intercanine retainer made of round 0.028-in stainless steel wire was used in the mandibular arch. 

## RESULTS

**Figure 7 -:**
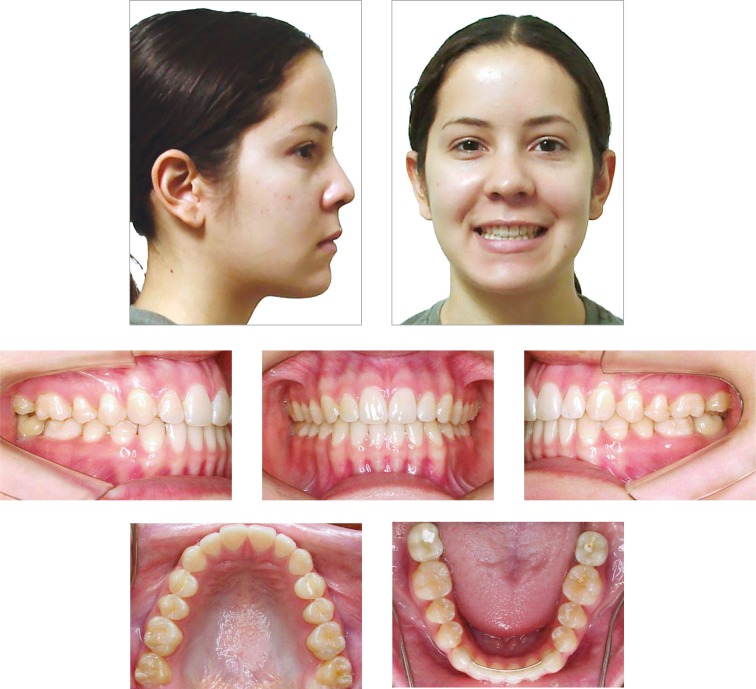
Final facial and intraoral photographs

**Figure 8 -:**
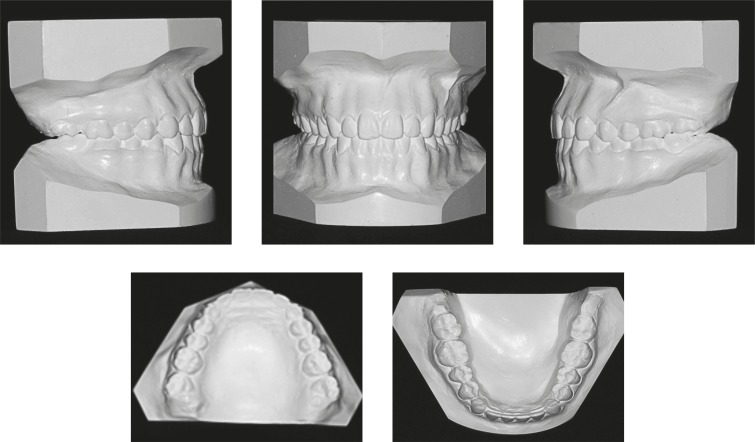
Final casts.

**Figure 9 -:**
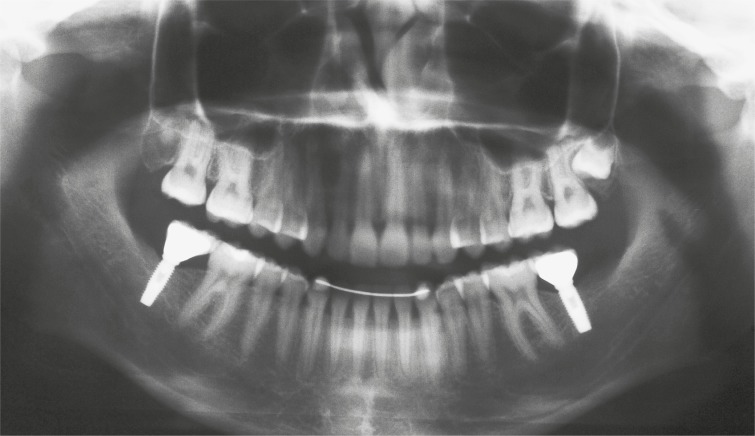
Final panoramic radiograph.

**Figure 10 -:**
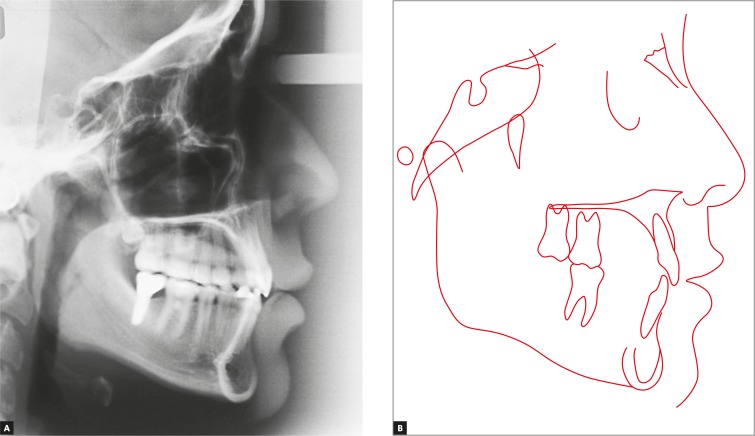
Final profile cephalometric radiograph (A), and final cephalometric tracing (B).

In assessing the patient's final records (Figs 7-10) it is clear that all intended objectives were achieved. Given that the patient did not grow during this period and no significant changes were implemented in the anterior region, only subtle facial changes were noted. In correcting the dental problems, such as intrusion of maxillary second molars and correction of the curve of Spee, the occlusal plane was leveled. Ideal occlusion was achieved between canines and molars at the expense of distal migration of maxillary teeth, and especially the mesial migration of mandibular teeth, particularly on the right side. Correction of maxillary and mandibular canine asymmetry in the anteroposterior direction, midline deviations, extrusion of mandibular second molars, reduced overbite, closing of mandibular spaces, and rotations were all solved in stages by means of specific mechanics.

**Figure 11 -:**
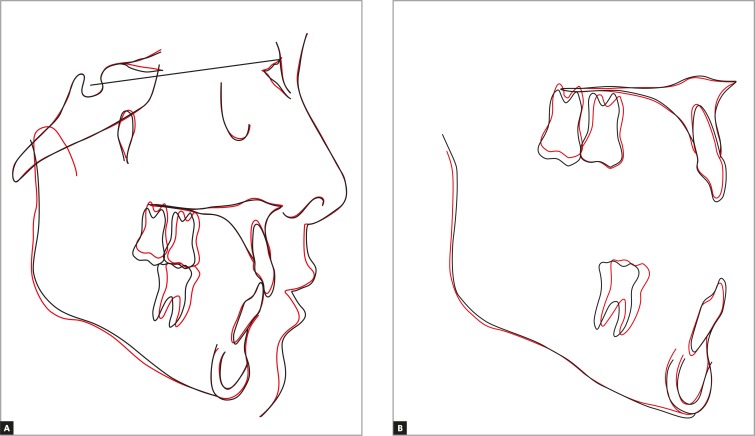
Total (A) and partial (B) superimpositions of initial (black) and final (red) cephalometric tracings.

By correcting deep overbite, the mandible was probably moved to a more anterior position, thus contributing to a mild improvement in facial harmony and providing, cephalometrically, a slight decrease in the value of the ANB angle (Steiner) from 2 to 1.5° (Table 1).

## FINAL CONSIDERATIONS

As previously mentioned, treatment was deliberately delayed by approximately eight months. However, it was later found that this delay should have lasted longer, given that in the final phase, in agreement with the implant dentist, it proved more advisable to wait another six months before performing surgery, which increased treatment time unnecessarily. On the other hand, delaying the process might probably mean increased extrusion of maxillary second molars.^5^^-^ 11

Despite treatment time increase, patients and legal guardians were very pleased with the end result, especially with regard to pleasant smile and balanced face. The goals initially set were met especially thanks to proper planning and use of biomechanical and rehabilitation resources based on individualized and thorough diagnosis as required by all atypical cases. Treatment of these cases should not follow predetermined classical protocols, but rather prompt professionals to hone their diagnostic skills in planning and carrying out a treatment tailored to suit individual peculiarities.
